# Giant Retroperitoneal Mucinous Tumor Supportively Diagnosed as a Dedifferentiated Liposarcoma by Fluorescence In Situ Hybridization of *MDM2* Gene

**DOI:** 10.5402/2011/261735

**Published:** 2011-06-16

**Authors:** Taku Naiki, Shuzo Hamamoto, Noriyasu Kawai, Aya Naiki-Ito, Yoshiyuki Kojima, Takahiro Yasui, Keiichi Tozawa, Kenjiro Kohri

**Affiliations:** ^1^Department of Nephro-urology, Graduate School of Medical Sciences, Nagoya City University, Mizuho-ku 467-8601, Nagoya, Japan; ^2^Department of Experimental Pathology and Tumor Biology, Graduate School of Medical Sciences, Nagoya City University, Mizuho-ku 467-8601, Nagoya, Japan

## Abstract

Surgical resection was performed on a 47-year-old woman for a retroperitoneal mass that weighed 8.5 kg. Histological examination revealed a myxoid sarcomatous tumor. Because diagnosis could not be determined by immunohistochemistry, attention was focused on *MDM2* (murine double minute) gene amplification by fluorescence in situ hybridization (FISH) analysis. The tumor was finally determined to be a dedifferentiated liposarcoma. We experienced a case of a giant retroperitoneal dedifferentiated liposarcoma. FISH analysis was useful for the diagnosis and determination of the therapeutic strategy.

## 1. Introduction

Liposarcoma is the most common of the soft tissue sarcomas encountered in adulthood [1]. Liposarcomas are generally found in the extremities, retroperitoneum, and in inguinal lesions. The clinical characteristics of liposarcomas closely reflect their pleomorphic histologies, and the large size lesions are more common in the retroperitoneum [2, 3]. Herein, we report a case of a dedifferentiated liposarcoma that weighed 8.5 kg for which FISH analysis contributed to the diagnosis.

## 2. Case Report

**Figure 1:**
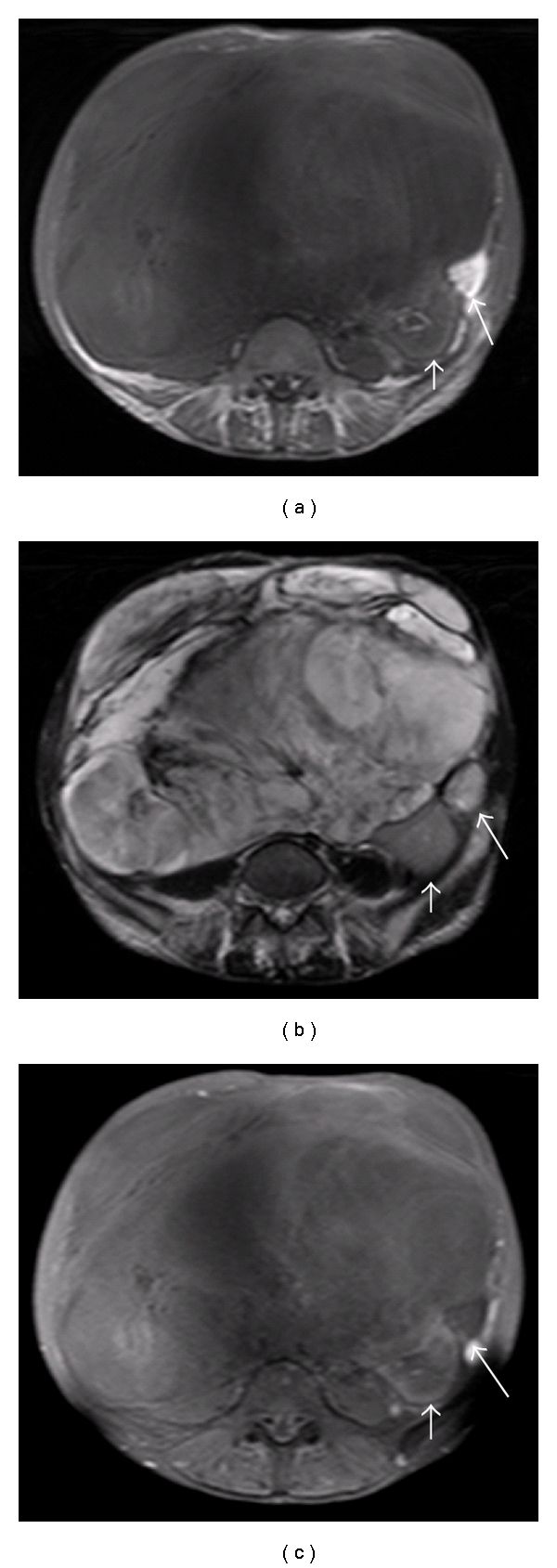
(a) shows T1-weighted images, (b) shows T2-weighted images, and (c) shows T1-weighted and fat suppression images on MRI. The giant tumor was T1 low intensity and T2 high intensity, but the small tumor was T1 high intensity, and with a changed low signal in fat suppression (long arrow). The right kidney was involved and pushed aside (small arrow).

**Figure 2:**
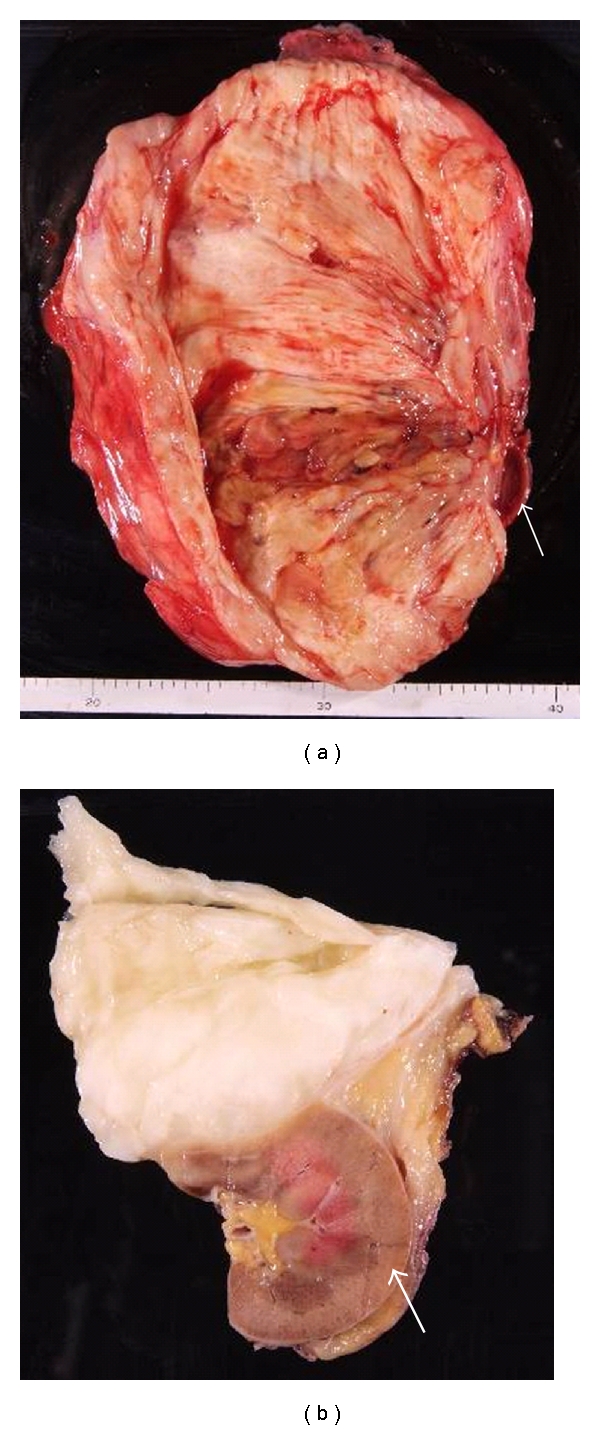
(a) shows resected mass and (b) shows two component of tumor as determined by CT and MRI after fixation. Macroscopically, the resected mass weight was 8.5 kg. The giant tumor was white, and the small one was yellowish (arrow: right kidney).

**Figure 3:**
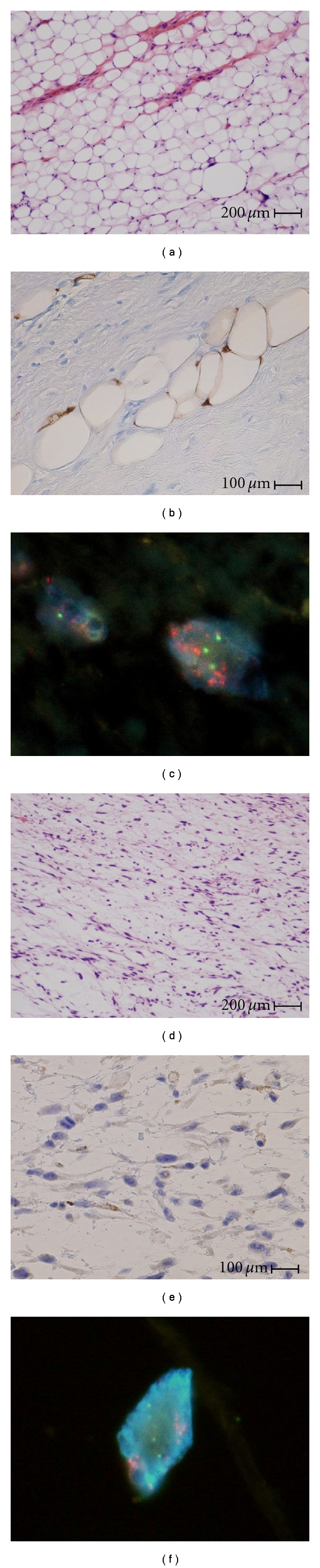
(a)–(c) show the high-power-magnification microscopic specimen of the small tumor, and (d)–(f) show those of the giant tumor. The two tumors had different expressions of S-100 protein (b), (e), but many *MDM2* red signals were recognized in nuclei of both tumors (c), (f) (Green: chromosome 12).

A 47-year-old woman was admitted to the Nagoya City University Hospital, Nagoya, Japan, complaining of an abdominal swelling that had been present for the past 12 months. Her abdomen was markedly swollen and felt hard without tenderness. Peripheral blood examination revealed slight anemia and slight elevation of C-reactive protein. No other disorders, including any tumor markers, were detected. A computed tomography scan (CT) showed two masses in the retroperitoneum, one of which was 26 × 15 × 29 cm and enhanced slightly, and the right kidney was involved and pushed aside. Almost all retroperitoneal cavities were filled with the giant tumor. The other mass was walnut-sized tumor adjacent to the large one, and mostly of low density. Magnetic resonance imaging (MRI) revealed the giant tumor was T1-low intensity and T2-high intensity, but the small tumor was T1-high intensity, with a changed low signal in fat suppression (Figure 1). This indicated that the small tumor was mainly fat-containing, and the giant tumor appeared to be mainly composed of mucinous tissue. Under general anesthesia, the tumor was excised with the right kidney en bloc. There was not much adhesion to surrounding tissues. Upon gross examination, the tumor was well circumscribed and encapsulated and weighed 8.5 kg (Figure 2). The tumor had two components as determined by CT and MRI. The two components were very clearly divided. The small one was yellowish, and histopathologically, they were mainly composed of fat cells that varied in size, that is, mature-appearing adipose tissue with scattered lipoblasts exhibiting large hyperchromatic nuclei. Immunohistochemical analysis revealed that the cells were strongly stained by S-100 protein which is a marker for fat (Figure 3). Therefore, this small tumor was diagnosed as a well-differentiated liposarcoma. On the other hand, giant tumor was white and, histopathologically, mainly contained abundant mucinous mesenchyme and filled with atypical spindle cells. There was extensive in-depth invasion into the renal parenchyma, but the surgical margin was negative. According to immunohistochemical analysis, the poorly differentiated tumor cells had no expression of several mesenchymal markers containing S-100 protein. Therefore, a comorbid malignant fibrous histiocytoma (MFH), or other myxoid tumor could not be ruled out. Consequently, we focused attention on FISH analysis, using specific probes for the *MDM2* gene (Figure 3). The amplification of *MDM2* gene was detected in the nuclei of both small and giant tumors. Thus, a definitive diagnosis was made of the two tumors as a dedifferentiated liposarcoma. The patient was strictly observed without adjuvant therapy. During the last follow-up investigation, performed 12 months after surgical intervention, the patientbecame well with no clinical or radiological signs of recurrence. 

## 3. Discussion

Liposarcomas are histologically defined as tumors composed of lipoblasts. They are currently classified into five groups: myxoid liposarcomas, well-differentiated liposarcomas, round cell liposarcomas, pleomorphic liposarcomas, and dedifferentiated liposarcomas. Dedifferentiated liposarcomas are characterized by the coexistence of well-differentiated and poorly differentiated, nonlipogenic areas in a portion of the same tumor or in the primary tumor and the recurrent tumor [4]. However, in this case, the two tumors were clearly divided and expression of S-100 protein was not detected in the poorly differentiated component. Therefore, we could not rule out the coexistence of a well-differentiated liposarcoma and MFH. Dedifferentiated liposarcomas have been a challenge to distinguish from other high-grade sarcomas, and some reports have described that most of MFH developing in the retroperitoneum are dedifferentiated liposarcomas [5, 6]. Recently, well-differentiated liposarcoma/atypical lipomatous tumors and dedifferentiated liposarcomas have been shown by cytogenetics to harbor ring and giant marker chromosomes consisting of amplicons of the 12q13–15 region, resulting in amplification of several genes, including most notably *MDM2* [7]. In addition, the prognosis of lipomatous tumors has been recently clarified on the basis of the genetic background [8]. There is little precise information as to the effectiveness of various therapies, but radical excision is the treatment of choice for liposarcomas, especially in dedifferentiated liposarcomas expressing the *MDM2* gene [3]. Some reports described postoperative radiation as a valuable adjuvant to surgical therapy, especially for the myxoid type, but the efficacy remains to be established [9]. Few reports have described the effectiveness of adjuvant chemotherapy. To our knowledge, this case represents the second largest primary retroperitoneal dedifferentiated liposarcoma that has been reported in the English literature [10]. Complementary molecular testing may refine the therapeutic strategy and result in avoiding ineffective adjuvant therapy.
